# High-Throughput Sequencing Indicates Novel *Varicosavirus*, *Emaravirus*, and *Deltapartitivirus* Infections in *Vitis coignetiae*

**DOI:** 10.3390/v13050827

**Published:** 2021-05-03

**Authors:** Tomoyuki Nabeshima, Junya Abe

**Affiliations:** 1 Department of Agriculture, Yamagata University, Tsuruoka 997-8555, Japan; 2 Smart Agriculture Division, JFE Engineering Corporation, Yokohama 230-8611, Japan; abe-junya@jfe-eng.co.jp

**Keywords:** next-generation sequencing (NGS), virome, RNA virus, grape

## Abstract

*Vitis coignetiae* samples were collected from several locations in the northern area of Japan, and virome analysis using a high-throughput sequencing technique was performed. The data indicated that some of the collected samples were in mixed infections by various RNA viruses. Among these viruses, three were identified as newly recognized species with support of sequence identity and phylogenetic analysis. The viruses have been provisionally named the Vitis varicosavirus, Vitis emaravirus, and Vitis crypticvirus, and were assigned to the genus *Varicosavirus*, *Emaravirus*, and *Deltapartitivirus*, respectively.

## 1. Introduction

Recent developments in high-throughput sequencing (HTS, or Next-Generation Sequencing (NGS)) technologies and bioinformatic analyses have resulted in advances in detecting viruses in plants [1,2]. Using HTS technologies, increasing numbers of studies describe novel viral species or strains, complex virome status in planta, or geographical distribution of viruses. These studies contribute to the understanding of viral ecology and evolution, pathogenicity, hosts, and epidemiology [1,2]. Additionally, these technologies are progressively reaching practical fields, such as diagnostic [3] or quarantine regulations [4].

The genus *Vitis* comprises more than 60 species. The most well-known and economically important *Vitis* species is the *Vitis vinifera* subsp. vinifera, which is the western Eurasian species. Vinifera’s ancestor is the wild *Vitis vinifera* subsp. sylvestris, naturally occurring in Europe, the Middle East, and Northern Africa [5]. Because cultivated grapes are perennial crops, and their propagation is mainly dependent on vegetative propagation, viral diseases pose considerable problems. Many researchers have been focusing on viral diseases in grapes, and more than 80 species of viruses have been described [6]. However, reports on viral infection in wild *Vitis* are elusive. Similarly, up to 30 grapevine species are native to East Asia and North America. Among these, the crimson glory vine (*V. coignetiae*) is native to the East Asian Pacific Refuge [5]. They naturally grow in areas of cool temperature and are distributed in Russia, Southern Kurile Islands, and Japanese Islands. In Japan, wild *V. coignetiae* is commonly found throughout the Japanese Islands, and they are also cultivated for fresh fruit, jams, juices, dried fruits, and production of wine [7]. In Korea, they are also used as a Korean folk medicine to treat various diseases, such as cancer and inflammatory disorders [8].

In this study, we performed an HTS analysis using *V. coignetiae* samples collected in Japan. The sequence data were then used to analyze viral sequences, from which we identified three novel RNA viruses.

## 2. Materials and Methods

### 2.1. Plant Materials and RNA Extraction

In September 2020, wild *Vitis coignetiae* leaf samples from several locations in the northern area of Japan (Table 1) were collected. Samples were stored at −80 °C until extraction. Frozen samples were also homogenized using a freeze crusher µT-48 (Taitec, Koshigaya, Japan) with supplied stainless beads. Total RNA was extracted using ISOSPIN Plant RNA with an assist buffer (Nippongene, Tokyo, Japan) and according to the manufacturer’s instructions.

### 2.2. HTS

RNA samples were bulked into two groups: 15 leaf samples (including three symptomatic leaves) from Hokkaido (hereafter called pool A for convenience) and the other 19 samples from Hokkaido and the North-East area of mainland Japan (pool B). Sequentially, bulked RNA was subjected to the construction of a cDNA library using the MGIEasy RNA Directional Library Prep Set (MGI Tech Co Ltd., Shenzhen, China). The DNA nanoballs (DNBs) were then prepared using the DNBSEQ-G 400 RS HTS Kit (MGI Tech Co., Ltd.), after which the generated DNBs were sequenced using DNBSEQ-G400 ((MGI Tech Co Ltd.) under 2 × 100 bp conditions. We obtained 38,507,344 and 35,823,926 reads from the pools A and B, respectively. Trimming of reads by their quality and *de novo* assembly was also performed using the CLC Genomic Workbench software (ver. 8.5.1; Qiagen, Hilden, North Rhine-Westphalia, Germany). Contigs shorter than 500 nt were discarded. To extract exogenous sequences, trimmed reads were mapped onto the *V. vinifera* genome (12×, GenBank assembly accession: GCA_000003745.2); then, unmapped sequences were collected before the de novo assembly. The aforementioned procedure resulted in 2260 and 1444 contigs from pools A and pool B, respectively, which were then used for subsequent analysis.

### 2.3. Analysis and Comparison of Viral Genome and Encoded Proteins

BlastN, BlastX, and BlastP analyses were performed at the National Center for Biotechnology Information (NCBI) against the database set for “viruses” (taxid: 10239). All the obtained contigs (>500 nt) were then subjected to BlastN and BlastX analyses. Annotations of subjects up to 100 were checked seeking for viral proteins (genome sequences) one by one, after which candidate contigs were selected for further studies. The ORF finder at NCBI was also used to find open reading frames (ORFs) in the candidate viral genomes. A minimal ORF length of 300 nt was considered. Protein motifs were also identified using the MOTIF finder at the Kyoto University Bioinformatics Center (https://www.genome.jp/tools/motif/, accessed on 6 April 2021) against the Pfam database. Alignment and phylogenetic analyses of the expected amino-acid sequences of viral proteins were also performed using MEGAX software (v10.2.2) [9]. Multiple sequence comparison by Log-Expectation was used for the alignment of amino-acid sequences, whereas the protein alignment tool was implemented using the CLC Genomic Workbench software to create visual images of the amino-acid sequence alignments and identical positions. For constructing a phylogenetic tree, the maximum likelihood method based on the JTT matrix-based model [10] with 1000 bootstrap replications was conducted. The percentage of nucleotide and amino-acid sequence identity was analyzed using a needle in EMBOSS (https://www.ebi.ac.uk/Tools/psa/emboss_needle/, accessed on 6 April 2021) between identified viruses and related viruses. In silico, protein analyses were also performed using the following software: NetNGlyc 1.0 server, http://www.cbs.dtu.dk/services/NetNGlyc/ (accessed on 6 April 2021) for predicting glycosylation sites; TMHMM server v. 2.0, http://www.cbs.dtu.dk/services/TMHMM/ (accessed on 6 April 2021), transmembrane helices; and SignalP 4.1 server, http://www.cbs.dtu.dk/services/SignalP-4.1/ (accessed on 6 April 2021), signal peptides.

### 2.4. RT-PCR

Reverse transcription (RT) was conducted in a 10-µL-volume containing 50 units of ReverTra Ace (Toyobo, Osaka, Japan), 1.0 µL RT buffer, 1 mM dNTPs (10 mM), 20 U RNase inhibitor (Toyobo), and 1.0 µM 6 mer random primer (Random Primer (hexadeoxyribonucleotide mixture; pd (N)6); TakaRa, Kusatsu, Japan). Total RNA was added to the reaction mixture to arrive at a final concentration of 10 to 20 ng·μL^−1^, after which the mixture was incubated at 42 °C for 30 min and 99 °C for 5 min. The RT product was then added to a 9.0 µL of the PCR mixture to obtain a final volume of 10 μL. RT-PCR was performed in a 10 µL mixture containing 0.25 U of the Blend taq polymerase (Toyobo), the reaction buffer for the Blend taq polymerase, 0.2 mM dNTPs, and 0.2 µM of forward and reverse primers. The primers used for each experiment are listed in Table S1. The RT-PCR products were then separated by electrophoresis on a 1.5% agarose gel and visualized by ethidium bromide staining.

### 2.5. Inoculation Test

Several attempts were made to transmit viruses from infected *V. coignetiae* to *Nicotiana benthamiana* and *Solanum lycopersicum* cv. Micro-tom. Mortar and pestle were used to grind infected leaf samples (500 mg) with 1 mL of 0.1 M phosphate (pH 7.2), 0.1 M MES (pH 5.5), or 0.1 M HEPES (pH 7.8) buffer containing 20 mM Na_2_SO_3_, 10 mM Na-DIECA, and 5 mM Na-EDTA. The resulting inoculum was then inoculated into the first true leaf of a two-week-old seedling of *N. benthamiana*, and a one-week-old seedling of *S. lycopersicum* through mechanical rubbing with carborundum, after which plants were grown in a growth chamber (25 °C, 16 h Photoperiod, 36 µmol·m^−2^·s^−1^. PPFD provided by a white fluorescent lamp). One month after inoculation, uninoculated upper leaves were collected, and total RNA was extracted as described earlier. Then, RT-PCR was conducted to check for viral infection, as described earlier.

## 3. Results and Discussion

### 3.1. Identification of a Varicosavirus-Like Virus

The genus *Varicosavirus* belongs to the family *Rhabdoviridae*, which classifies viruses with a diverse host range, including vertebrates, invertebrates, and plants [11]. The genome of the *Rhabdoviridae* consists of single-stranded (ss) negative-sense RNAs. At present, only one *Varicosavirus*, the lettuce big-vein associated varicosavirus (LBVaV) [12], has been listed by the International Committee on Taxonomy of Viruses (ICTV; https://talk.ictvonline.org/, accessed on 17 December 2020). Similarly, two tentative species, red clover-associated varicosavirus (RCaVV), and Alopecurus myosuroide varicosavirus 1 (AMVV1) have been reported [13,14]. All the aforementioned reports describe the genome of the *Varicosavirus* as a two-segmented RNA type.

Results of the BlastX search in this study are summarized in Table 2. Contig 38 in pool A (38A) indicated a 39.6% identity with LBVaV (QBC40942.1). Additionally, the nucleotide sequence of 38A indicated a 64.0% identity with a complete genome sequence of the RNA1 segment of RCaVV, as indicated by BlastN. Another 5535 nt contig (85A) had the highest identity with the RNA2 segment of Rhabdovirus YHG-2013 (35.2%), followed by LBVaV (QBC40942.1; 28.9%) and AMVV1 (YP009130617.1, designated as a “Black grass varicosavirus-like virus” in the database; 27.0%). It was, therefore, suggested that the two-segmented varicosavirus-like viruses be included in pool A. Thus, we provisionally named this putative virus as Vitis varicosavirus (VVV). Subsequently, we designed specific primer sets (Table S1) according to sequences of 38A and 85A contigs and conducted RT-PCR to confirm the VVV presence in *V. coignetiae* samples. The result indicated that two pool A samples were VVV-positive (Table 1). Partial sequences of RNA1 and RNA2 of VVV were then deposited in GenBank as LC604719 and LC604720, respectively.

**Figure 1:**
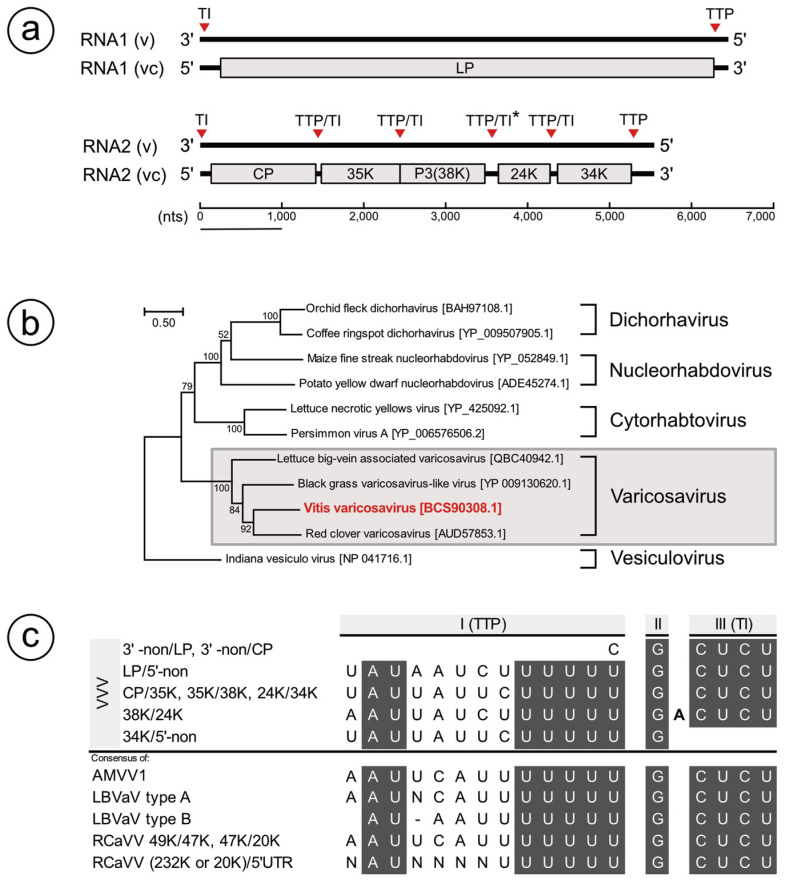
(**a**) Proposed genome organization of Vitis varicosavirus (VVV). In the negative-sense genome (v), positions of identified transcription termination/polyadenylation (TTP) sites and transcription initiation (TI) sites are shown as red triangles. The asterisk indicates an additional A in the TI/TTP site. In the positive-sense strands (vc), open reading frames are presented as gray boxes. LP: Large protein, CP: Coat protein. (**b**) A phylogenetic tree showing the deduced amino-acid sequence of the partial LP gene of VVV showing its position with members of the genus *Varicosavirus*. The evolutionary history was inferred using the maximum likelihood method and the JTT matrix-based model. The tree with the highest log likelihood (−53,228.85) is shown. The percentage of trees in which the associated taxa clustered together is also shown next to the branches. Initial tree(s) for the heuristic search were obtained automatically by applying Neighbor-Join and BioNJ algorithms to a matrix of pairwise distances estimated using the JTT model and then selecting the topology with superior log likelihood values. The tree is drawn to scale, with branch lengths measured in the number of substitutions per site. There were a total of 2308 positions in the final dataset. Indiana vesiculo virus was used as an outgroup. (**c**) Comparison of TI/TTP sites among VVV, LBVaV, and RCaVV. Conserved motifs were shown as gray boxes with white letters. II: intergenic region.

ORF search in the proposed VVV partial genome identified one ORF in RNA1 and five ORFs in RNA2 (Figure 1a). In RNA1, the 2008 amino-acid (aa) protein with the expected molecular mass of 227 kDa, which had an RNA-dependent RNA polymerase (184–1057, Pfam: PF00946, 1.6 × 10^−161^), mRNA-capping region V (1071–1287, Pfam: PF14318, 7.2 × 10^−18^), and virus-capping methyltransferase (1618–1664, Pfam: PF14314, 0.19) motifs, was identified. The function of this 227.2 kDa protein was proposed to be equivalent to that of the L-protein (LP) of other members of the *Varicosavirus* members [12,13,14].

Alternatively, in the proposed partial RNA2 genome, five ORFs encoding 425aa (48 kDa), 319aa (35 kDa), 344aa (38 kDa), 209aa (24 kDa), and 299aa (34 kDa) proteins (Figure 1a) were identified. The presence of these five ORFs in RNA2 of VVV was consistent with that of LBVaV [15], whereas RNA2 of RCaVV and AMVV1 had three ORFs [13,14]. The 48 kDa protein shared a 47% identity with the nucleoprotein (a homolog of coat protein (CP)) of LBVaV (Q91QN9.1), whereas the 38 kDa protein shared a 29% identity with Protein 3 of LBVaV (Q68Y30.1). Contrarily, BlastP analysis returned no significant results for the 35 kDa, 24 kDa, and 34 kDa proteins, whereas the MOTIF finder analysis also detected no motif in these three proteins (cutoff E value ≤ 0.1). Thus, the function of these proteins remains uncharacterized.

Phylogenetic analysis based on the LP amino-acid sequences also supported the assignment of VVV into the *Varicosavirus*. Consistent with previous studies [14], AMVV1 and RCaVV made a subcluster within the *Varicosavirus* as well; thus, VVV was placed in this subcluster (Figure 1b). Similarly, CP of VVV showed the closest identity with that of RCaVV but was also close to that of LBVaV (Table 3). Therefore, when one considers the number of ORFs in RNA2 and the presence of putative homologous protein 3 in VVV and LBVaV but not in RCaVV and AMVV1, evolutional relations between these viruses are confusing. Further identification of novel *Varicosavirus* species should solve this situation.

In the genome sequences of RNA1 and RNA2 of VVV, conserved gene junction sequence, which may be involved in transcription termination/polyadenylation (TTP) of upstream gene and transcription initiation (TI) of a downstream gene [11,16], was identified (Figure 1c). These junctions were located even up/downward of three uncharacterized genes. The sequences of the junctions matched well with those of AMVV1, LBVaV, and RCaVV, only despite insertion of A between the intergenic and TI regions within 38 KDa/24 KDa junctions (Figure 1c).

Based on the foregoing results, we propose that VVV is a new member of the genus *Varicosavirus*, in which two-segmented genomes encode the putative LP, CP, and four uncharacterized proteins.

### 3.2. Identification of an Emaravirus-Like Virus

The genus *Emaravirus* is the only clarified genus in the *Fimoviridae* family [17]. Since the first description of the European mountain ash ringspot-associated Emaravirus (EMARaV) in *Sorbus aucuparia* in 2007 [18], increasing numbers of *Emaravirus* have been reported [19]. The current ICTV taxonomy lists 11 species in *Emaravirus*, which are prevalent in fruit or ornamental trees and shrubs. The genome of *Emaravirus* also consists of four to eight segments of negative-sense ssRNAs. They are not capped or polyadenylated, and they contain 18–20 nt complementary sequences at the 5′ and 3′ termini [19].

Using BlastX analysis, we found that some obtained contigs encoded proteins, which indicated some homology to that of *Emaravirus* proteins. Then, we used the *Emaravirus* genome dataset at NCBI (taxid: 675845) for customized local BlastX to detect emaravirus-like RNAs. All obtained contigs were then used as a query. Results indicated seven emaravirus-like contigs from pool A and five from pool B (Table 2). We also designed primer sets and checked the presence of these RNAs in *V. coignetiae* using RT-PCR. The results indicated that five and one samples, respectively, in pool A and B were positive. Note that in the positive samples, these five RNA segments could be detected without chipping (Figure S1). Furthermore, the sequencing of RNA3 and RNA4 amplicons from pool A revealed that there was a combination of 2a and 4a in no. 4, 5, and 6, as well as a combination of 2b and 4b in sample no. 17 (data not shown). From these, it was considered that the emaravirus-like virus, comprising at least five RNA segments, was present in these samples. Thus, we named this RNA set Vitis emaravirus (VEV). The proposed partial genome sequences were also deposited in GenBank (Table 1). In the following sections, we will focus on the genome set (1b, 2b, 3c, 4c, and 6b) recovered from pool B.

**Figure 2:**
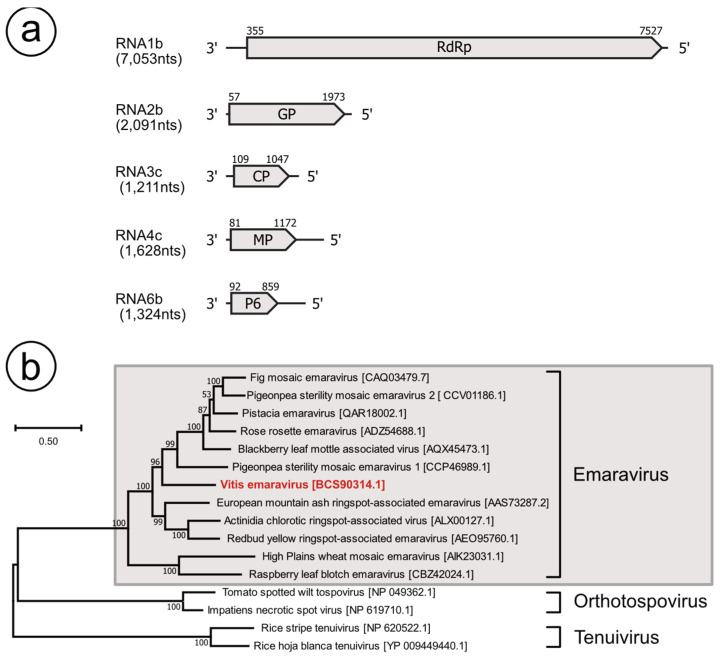
(**a**) Proposed genome organization of Vitis emaravirus (VEV). Genome segments of the negative-sense RNA virus are displayed as mRNAs with the encoded open reading frames as gray arrows. RdRp: RNA-dependent RNA polymerase, GP: glycoprotein precursor, CP: coat protein, MP: movement protein. (**b**) Phylogenetic tree showing the deduced amino-acid sequence of the partial RdRp gene of VEV showing its position with members of the *Emaravirus* genus. The evolutionary history was inferred using the maximum likelihood method and the JTT matrix-based model. The tree with the highest log likelihood (−71,655.15) is also shown. The percentage of trees in which the associated taxa are clustered together is shown next to the branches as well. Initial tree(s) for the heuristic search were obtained automatically by applying Neighbor-Join and BioNJ algorithms to a matrix of pairwise distances estimated using the JTT model and then selected using the topology with a superior log likelihood value. The tree is drawn to scale, with branch lengths measured in the number of substitutions per site. There were 3370 positions in the final dataset.

ORF search within obtained segments of the VEV genome is summarized in Figure 2a. RNA1b (311B) encodes a 2300aa protein with an estimated molecular mass of 269 kDa, which had a 48.9% identity with RdRp of the aspen mosaic-associated virus (CAA0079389.1), a provable member of the *Emaravirus* family, found in *Populus tremula* [20]. Motif analysis within this protein was identified as a Bunyavirus RdRp motif (646–1389; Pfam: PF04196; 3.8 × 10^−54^), suggesting that this protein was orthologous to the conserved RdRp members of *Fimoviridae*. Similarly, RNA1a (201A) encodes the 2300aa protein, which has a 97.4% identity with that of 311B, and the Bunyavirus RdRp motif (Pfam: PF04196) that was conserved.

RNA2b (179B) encoded a putative glycoprotein precursor (GP) in length 638aa (73 kDa), which had 43.0% identity with GP of pigeon-pea-sterility mosaic emaravirus 2 (PPSMV2; QBA83608), a provable member of *Emaravirus* found in *Cajanu cajin* [21]. In silico analysis of putative GP indicated three putative transmembrane helices at positions aa 5–27, 174–191, and 581–603 and five predicted N-glycosylation sites at aa 69, 205, 243, 332, and 439. We could not detect potential cleavage sites using SignalP analysis. However, the tetra peptide ADDN_193–196_ was present, which might produce 22 kDa (G_n_) and 50 kDa (G_c_), as described in other emaraviruses [18,22,23]. RNA2a (69A) encodes 638 aa putative GP, which is 98.10% identical to that in RNA2b. In silico analyses returned similar results.

RNA3c (58B) encoded a 312aa protein (35 kDa), which had a 44.1% identity with that of CP of the fig mosaic virus (FMV; CAA0079389.1), which is an approved member of *Emaravirus* found in *Ficus carica* [22,24]. In CPs of *Emaravirus*, two conserved amino-acid blocks (NX_2_SXNX_3_A and NXLA), which are potential nucleocapsid motifs have been reported [22,23,25]. The 35 kDa VEV protein was also posed as an NXLA_197–200_ motif, but instead of the NX_2_SXNX_3_A motif, EFVSFNKA_148–157_ was observed (Figure S2). Although there was a substitution of N by E, EFVSFNKA_148–157_, and putative nucleocapsid motif NXLA_197–200_, were aligned clearly along with those in other emaraviruses. Therefore, we considered that 35 kDa was that of CP of VEV. Similarly, RNA3a (19A) encoded the 312aa protein, which is perfectly identical to that of RNA3c. Contig 574A was shorter than those of 19A and 58B, and ORF in length 270aa, which lacked 42 residues in the C termini was observed as well. Additionally, in 270aa, 10 residues were different from those of 312aa in 19A and 58B, but EFVSFNKA_148–157_ and NXLA_197–200_ motifs remained conserved.

Additionally, RNA4c encoded 363aa proteins (41 kDa) with 39.5% identity with the movement protein (MP) of PPSMV2 (ANQ90753.1). This 41 kDa was also recognized as belonging to the Emaravirus_P4 super family (42–361, Pfam: 16505, 1.1 × 10^−88^), thus sharing the MP function. The ORF finder also indicated that RNA4a and RNA4b encoded 343aa (39 kDa) identical proteins, which also posed as the Emaravirus_P4 super family motif. The 343aa in RNA4a and RNA4b showed high identity with the 41 kDa of RNA4c, and only two amino-acid changes were found. However, they lacked 17 amino-acid residues in the N terminus compared to that of the 41 kDa of RNA4c; thus, they were considered to be partial CDS of MP.

The last segment, 123B encodes 255aa proteins (29 kDa), having 25.3% identity with Protein 6 (P6) of pistacia emaravirus (PiVB; QAR18008.1), a provable member of *Emaravirus* found in *Pistacia* spp. [26]. Although the identity between this 29 kDa and P6 of PiVB was low, and the function of this 29 kDa protein could not be estimated by the MOTIF finder, P6 of PiVB was the only significant result obtained using BlastP (Query cover: 66%; E value: 1.0 × 10^−5^). Thus, we considered that this segment was homologous to RNA6 of PiVB. Amino-acid comparison between P6 among emaraviruses also indicated that VEV had an identity of 11.2–23.4% with known emaraviruses (Table 4). Contig 70A also encoded 255aa proteins, with an amino-acid sequence of 98.4% identity to that of 123B. With the fact that this putative RNA6 could be detected in VEV-positive samples but not in VEV negative samples (Figure S1), we concluded that it was a component of the VEV genome. The segment homologous to RNA5 of other emaraviruses could not be found in this study.

Tatineni et al. [27] demonstrated that the phylogenetic tree based on the RdRp sequence represented three subclusters within the *Emaravirus* genus. This picture was repeatedly confirmed by other studies [23,25,26,28]. Consistent with previous studies, our phylogenetic analysis depicted subclusters, including FMV, PPSMV2, PiBV, rose rosette emaravirus, blackberry leaf mottle-associated virus, PPSMV1 (designated as subclade A by Zheng et al. [25]), subcluster B, including EMARaV, actinidia chlorotic ringspot-associated emaravirus, and redbud yellow ringspot-associated emaravirus, subcluster C, including high plains wheat mosaic virus and raspberry leaf blotch emaravirus, in the *Emaravirus* genus (Figure 2b). VEV also created a new branch of subcluster A with a high-bootstrap value support. Amino-acid comparison of RdRp among the *Emaraviruses* supported that VEV had relatively higher identity, 47.7–49.1%, with members of subclusters A and B as well, compared to those of the other two viruses in subcluster C (Table 4). A similar tendency was observed for GP, CP, and MP.

Based on the foregoing results, we propose that VEV is a new member of the genus *Emaravirus*. VEV was consistent with at least five-segmented genomes, which encoded putative RdRp, GP, NP, P3, and one uncharacterized protein. Although the possibility of the presence of other segments of VEV could not be denied, the genome set of detected VEV satisfied the minimal composition of known emaraviruses.

### 3.3. Identification of Partitivirus-Like Virus

The genome of members of the *Partiviridae* family was consistent for two- to three-segmented double-stranded (ds) RNAs, which were encapsulated individually to make biparticulate virions [29]. Segments encoded by RdRp were also designated as dsRNA1, and the segment encoded by CP was designated as dsRNA2, respectively. The family has host ranges in fungi, plants, and protozoa. The current ICTV taxonomy also puts five genera in this family: *Alphapartitivirus*, *Betapartitivirus*, *Cryspovirus*, *Gammapartitivirus*, and *Deltapartitivirus*. At present, both plant and fungal viruses are listed in *Alphapartitivirus* and *Betapartitivirus*. Alternatively, *Gammapartitivirus* and *Deltapartitivirus* include only fungal and plant viruses, respectively [30,31]. Similarly, the *Cryspovirus* genus also currently contains only one protozoal virus, Cryptosporidium parvum virus 1, isolated from *Cryptosporidium* [32].

We found that 1563 and 1521 nt contigs from pool A (369A and 784A) and 1563 and 1521 nt contigs from pool B (567B and 146B) had some homology to the *Partitiviridae* genome (Table 2). Pairwise alignment by needle also indicated that the nucleotide sequence of 369A and 567B had a 95.8% identity, whereas 784A and 146B had 97.7% identities, respectively. It was, therefore, suggested that the partitivirus-like virus infected both pool A and B samples, but infecting strains in pools A and B, respectively, were different. We provisionally named this putative virus as Vitis cryptic virus (VCV) and proposed a partial genome sequence that we deposited in GenBank (Table 1).

**Figure 3:**
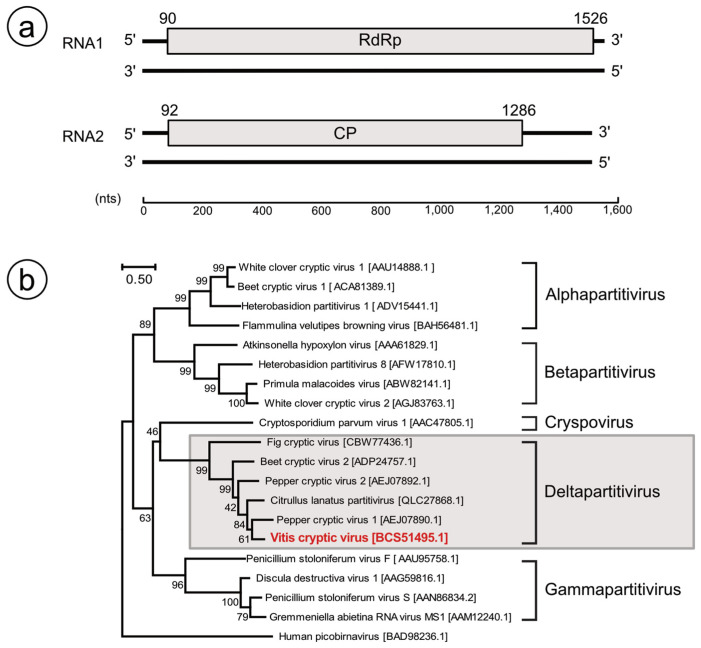
(**a**) Proposed partial genome organization of Vitis cryptic virus (VCV). Open reading frames are presented as gray boxes. RdRp: RNA-dependent RNA polymerase, CP: Coat protein. Numbers above gray boxes indicate start/end nucleotide positions of open reading frames. (**b**) Phylogenetic tree showing the deduced amino-acid sequence of the RdRp gene of VCV showing its position with members of the Deltapartitivirus genus. The evolutionary history was inferred using the maximum likelihood method and the JTT matrix-based model. The tree with the highest log likelihood (−23,194.31) is as presented. Percentages of trees in which the associated taxa clustered together are shown next to the branches. Initial tree(s) for the heuristic search were obtained automatically by applying Neighbor-Join and BioNJ algorithms to a matrix of pairwise distances estimated using the JTT model and then selecting the topology with superior log likelihood values. The tree is drawn to scale, with branch lengths measured in the number of substitutions per site. There were 841 positions in the final dataset. The human picobirna virus was used as an outgroup.

The proposed partial genome of VCV is as drawn in Figure 3a. The ORF search identified a single ORF in each segment. RNA1a (369A; LC602838) encoded a 478aa (54 kDa) protein with viral RNA-dependent RNA polymerase (38–454, Pfam: PF00680, 9.7 × 10^−72^) and viral RNA-directed polymerase (192–365, Pfam: PF02123, 1.4 × 10^−5^), which has a 66.7% identity with RdRp of the citrullus lanatus cryptic virus (CiLCV; APT68925.1), a provable member of *Deltapartitivirus* identified in watermelon [33,34]. This protein also has 65.7% identity with RdRp of pepper cryptic virus 1 (PepCV1; AYA43794.1), a type species of *Deltapartitivirus.* Similarly, RNA1b (567B; LC602840) posed one ORF, which encodes 478aa putative RdRp showing 95.8% identity with that of RNA1a.

RNA2a (784A; LC602839) encoded a 397aa protein (46 kDa), which had a 47.5% identity with CP of CiLCV (QLC27869.1). Then, alignment of this putative 46 kDa putative CP and CPs in the other five approved deltapartitivirus (FMV, PepCV1, PepCV2, beet cryptic virus 2 (BCV2), and BCV 3) were performed. Without the 46 kDa of VCV, amino acids were identical at 14 positions, and adding the 46 kDa protein to them reduced the identical points to 11 positions (data not presented). Low aa sequence identities among CPs in partitiviruses were reported previously [35,36], which suggests that partitivirus CPs had higher evolution rates than those in RdRps [35]. Similarly, RNA2b (146B; LC602841) posed one of the ORFs, which encodes the 397aa putative RdRp, showing 98.2% identity with that in RNA2a.

Phylogenetic analyses based on the RdRp amino-acid sequence also showed that this virus was clustered into the genus *Deltapartitivirus* (Figure 3b), in which all approved members are plant viruses. Comparison of the genome sequence and the amino-acid sequence of RdRps and CPs with other approved *Deltapartitivirus* and CiLCV and type species in *Alphapartitivirus*, *Betapartitivirus*, and *Gammapartitivirus* are presented in Table 5. The RdRp and CP of VCV also had the highest similarity with CiLCV, followed by PepCV1. Consistent with phylogenetic trees based on RdRps, alignment of CPs to VCV, PepCV1, PepCV2, and CiLCV indicated 74 identical positions and some conserved blocks, which could not be recognized with FMV, BCV2, and BCV3, as described earlier (Figure S3). These suggested relatively close relationships among VCV, PepCV1, PepCV2, and CiLCV in deltapartitiviruses.

Based on the foregoing results, we propose that VCV is a new member of the genus *Deltapartitivirus*, which is consistent with the two-segmented genome encoding RdRp and CP.

### 3.4. Mix Infection Status in V. coignetiae

BlastX search revealed two contigs having high homology with the grapevine Pinot gris virus (GPGV). Infection of GPGV (LC601811 and LC601812) in collected materials was confirmed previously [37]. Additionally, there are two contigs with high homology with the grapevine berry inner necrosis virus (GINV). A nucleotide sequence of 7106 nt (LC605829) and 7204 nt (LC605830) contigs also indicated 78.6% and 79.3% identity with reported GINV sequence (D88448.2), as indicated by BlastN. LC605829 poses of ORFs encoded RdRp, MP, and partial CP, whereas LC605830 poses completely that of RdRp, MP, and CP. BlastP analysis also indicated that RdRp and MP in LC605829 had an 84.4% and 86.3% identity, respectively, with those of known amino-acid sequences (YP_004293216.1 and YP_004293217.1), whereas RdRp, CP, and MP in LC605830 had 84.6%, 88.3%, and 94.4% identity with YP_004293216.1, YP_004293217.1, and APT43224.1. GPGV [38] and GINV [39] are members of the *Trichovirus* genus in the *Triviridae* family. Trichovirus has a monopartite, positive-sense ssRNA, which encodes up to six ORFs [40]. Among these, 34 samples were used, and 12 samples were mix-infected by up to four viruses (Table 1). Presented data indicate the mix infection status by a wide range of RNA viruses in wild *V. coignetiae* habited in Japan. There were no plant materials, which showed obvious symptoms with single viral infections. Thus, we could not characterize symptoms caused by these viruses in *V. coignetiae*. Pictures of observed symptomatic leaves are available in Figure S4. Since we collected all materials from natural woods in rural areas, apart from commercial vine fields, graft transmission of the virome from commercial grapevine was unlikely. It is presumed that the viruses most have become infected by a vector over time and the diversity is the result of recombination and mutations.

### 3.5. Inoculation Test

Based on previous studies on LBVaV [12], we prepared three types of inoculum using three different buffers. The leaf used for inoculation was sample no. 1, which was infected by VVV, GPGV, and GINV (Table 1). Regardless of the buffer used for preparation, the resulting inoculum did not induce infection of tested viruses to *S. lycopersicum* and *N. benthamiana* (data not presented).

## 4. Conclusions

HTS in *V. coignetiae* identified three novel viruses: Vitis varicosavirus, Vitis emaravirus, and Vitis cryptic virus. From our findings, we conclude that each virus should be assigned to the *Varicosavirus*, *Emaravirus*, and *Deltapartitivirus* genera, respectively. To the best of our knowledge, this is the first identification of these three genera in the *Vitis* family.

## Figures and Tables

**Table 1:** Summary of *Vitis coignetiae* sample information used in this study.

Pool	Sample No.	Location (Prefecture)	Symptom	Viral Infection ^a^				
				VVV	VEV	VCV	GPGV	GINV
A	1	45°07′46.59″ N, 142°12′06.04″ E (Hokkaido)	Mosaic	+	−	−	+	+
	2	45°07′44.85″ N, 142°12′05.18″ E (Hokkaido)	None	−	−	+	+	+
	4	45°07′42.63″ N, 142°12′02.99″ E (Hokkaido)	None	−	+	+	−	−
	5	45°07′41.91″ N, 142°12′02.86″ E (Hokkaido)	None	−	+	+	−	−
	6	45°07′40.39″ N, 142°12′02.21″ E (Hokkaido)	None	−	+	+	−	−
	7	45°07′43.23″ N, 142°12′03.69″ E (Hokkaido)	Mosaic	−	−	−	+	+
	8	45°07′46.74″ N, 142°12′03.09″ E (Hokkaido)	Mosaic	+	−	−	+	+
	9	45°07′49.68″ N, 142°11′24.80″ E (Hokkaido)	None	−	−	+	+	+
	11	44°57′27.96″ N, 142°13′19.85″ E (Hokkaido)	None	−	−	+	−	−
	12	44°52′55.93″ N, 142°12′43.34″ E (Hokkaido)	None	−	−	−	−	−
	13	44°56′25.57″ N, 142°13′28.72″ E (Hokkaido)	None	−	−	−	−	−
	14	44°56′15.11″ N, 142°13′18.44″ E (Hokkaido)	None	−	−	−	−	−
	15	44°53′35.22″ N, 142°12′10.86″ E (Hokkaido)	None	−	−	−	−	−
	16	44°53′33.27″ N, 142°12′06.99″ E (Hokkaido)	None	−	−	−	−	−
	17	44°53′30.92″ N, 142°12′07.58″ E (Hokkaido)	None	−	+	+	−	−
B	18	43°08′03.04″ N, 140°49′27.85″ E (Hokkaido)	None	−	−	+	−	−
	19	43°08′00.95″ N, 140°49′30.55″ E (Hokkaido)	None	−	+	+	−	−
	20	43°00′28.97″ N, 140°52′34.70″ E (Hokkaido)	None	−	−	−	−	−
	21	43°00′23.97″ N, 140°52′41.01″ E (Hokkaido)	None	−	−	−	−	−
	22	43°21′23.14″ N, 142°14′37.98″ E (Hokkaido)	None	−	−	−	−	−
	23	43°21′18.17″ N, 142°14′35.64″ E (Hokkaido)	None	−	−	+	−	−
	24	43°27′19.82″ N, 142°30′06.96″ E (Hokkaido)	None	−	−	+	−	−
	25	43°27′19.88″ N, 142°30′14.23″ E (Hokkaido)	None	−	−	−	−	−
	26	43°26′21.10″ N, 142°35′43.27″ E (Hokkaido)	None	−	+	+	−	−
	30	40°11′22.55″ N, 141°29′15.77″ E (Iwate)	Leaf roll	−	−	+	−	+
	31	40°07′45.35″ N, 141°31′08.13″ E (Iwate)	Leaf roll	−	−	+	−	+
	32	40°07′44.72″ N, 141°31′04.26″ E (Iwate)	Leaf roll	−	−	+	−	−
	33	40°07′45.17″ N, 141°30′59.01″ E (Iwate)	Mosaic	−	−	+	−	+
	34	39°00′18.48″ N, 140°51′42.33″ E (Iwate)	None	−	−	+	−	−
	35	39°00′15.42″ N, 140°51′35.98″ E (Iwate)	None	−	−	+	−	−
	36	39°59′03.19″ N, 140°43′08.54″ E (Akita)	None	−	−	−	−	−
	37	38°58′51.20″ N, 140°43′04.73″ E (Akita)	None	−	−	+	−	−
	38	39°00′55.83″ N, 140°38′36.00″ E (Akita)	None	−	−	+	−	−

^a^ Viral infection was determined by RT−PCR. VVV: Vitis varicosavirus, VEV: Vitis emaravirus, VCV: Vitis cryptic virus, GPGV: grapevine Pinot gris virus, GINV: grapevine berry inner necrotic virus. +/− indicates positive/negative virus.

**Table 2:** Assembled contigs and related RNA segments of the three viruses detected in this study.

Virus ^a^	Genomic Segments	Contig No. ^b^	Length (nt)	No. of Reads	Best Much in BlastX	Query Coverage (%)	Sequence Identity (%)	Accession Numbers (NCBI, GenBank)
VVV	RNA1	38A	6450	19,629	Lettuce big-vein associated varicosavirus [QBC40942.1]	90	39.6	LC604719
	RNA2	85A	5535	20,846	Black grass varicosavirus-like virus [YP009130617.1]	16	27.0	LC604720
VEV	RNA1a	201A	7083	11,122	Aspen mosaic-associated virus [CAA0079389.1]	96	49.3	LC604721
	RNA2a	69A	2099	20,739	Pigeonpea sterility mosaic emaravirus 2 [QBA83608.1]	91	43.1	LC604722
	RNA3a	19A	1209	7735	Fig mosaic emaravirus [AWS21340.1]	74	44.1	LC604723
	RNA3b	574A	881	308	Fig mosaic emaravirus [AWS21340.1]	91	44.4	LC604732
	RNA4a	184A	1270	574	Pigeonpea sterility mosaic emaravirus 2 [ANQ90780.1]	84	39.8	LC604724
	RNA4b	185A	1596	8948	Pigeonpea sterility mosaic emaravirus 2 [ANQ90780.1]	67	39.8	LC604725
	RNA6a	70A	1335	13,473	Pistacia emaravirus [QAR18008.1]	42	25.5	LC604726
	RNA1b	311B	7053	1149	Aspen mosaic-associated virus [CAA0079389.1]	97	48.9	LC604727
	RNA2b	179B	2091	1249	Pigeonpea sterility mosaic emaravirus 2 [QBA83608.1]	91	43.0	LC604728
	RNA3c	58B	1211	1149	Fig mosaic emaravirus [AWS21340.1]	74	44.1	LC604729
	RNA4c	76B	1628	2137	Pigeonpea sterility mosaic emaravirus 2 [ANQ90780.1]	65	39.5	LC604730
	RNA6b	123B	1324	3090	Pistacia emaravirus [QAR18008.1]	38	25.3	LC604731
VCV	RNA1a	369A	1563	1843	Citrullus lanatus cryptic virus [APT68925.1]	90	66.7	LC602838
	RNA2a	784A	1521	937	Citrullus lanatus partitivirus [QLC27869.1]	77	47.0	LC602839
	RNA1b	567B	1563	1349	Citrullus lanatus cryptic virus [APT68925.1]	90	65.6	LC602840
	RNA2b	146B	1521	746	Citrullus lanatus partitivirus [QLC27869.1]	77	47.3	LC602841

^a^ VVV: Vitis varicosavirus, VEV: Vitis emaravirus, VCV: Vitis cryptic virus. ^b^ Alphabets A and B that follow numbers indicate that the contig is pool A-derived and pool B-derived, respectively.

**Table 3:** Percent identity of the amino-acid sequence of the Vitis varicosavirus proteins with other varicosaviruses.

	Vitis Varicosavirus (VVV)		
	LP ^a^	CP ^a^	P3 ^a^
Red clover varicosavirus (RCaVV)	40.9	30.1	-
Lettuce big-vein-associated varicosavirus (LBVaV)	39.6	27.3	29.0
Alopecurus myosuroides varicosavirus 1 (AMVV1)	38.6	27.0	-

^a^ GenBank accessions AUD57853.1, QBC40942.1, and YP_009130620.1 were used for a large protein (LP) of RCaVV, LBVaV, and AMVV1. GenBank accessions AUD57854.1, AAU12861.1, and YP_009130617.1 were used for coat protein (CP) of RCaV, LBVaV, and AMVV1. GenBank accession AFA36173.1 was used for Protein 3 (P3) of LBVaV. BlastP analysis returned only P3 of LBVaV for significant results when VVV P3 was used as a query.

**Table 4:** Percent identity of amino-acid sequence of *Vitis emaravirus* with other emaraviruses.

Virus	Vitis Emaravirus (VEV)				
	RdRp [GenBank]	GP [GenBank]	CP [GenBank]	MP [GenBank]	P6 [GenBank]
Pistacia emaravirus	48.9 [QAR18002.1]	39.3 [QAR18003.1]	38.6 [QAR18004.1]	35.0 [QAR18005.1]	22.6 [QAR18008.1]
Pigeonpea sterility mosaic emaravirus 2	48.6 [CCV01186.1]	42.1 [YP_009268865.1]	40.3 [YP_009268864.1]	37.4 [YP_009268866.1]	21.0 [YP_009268862.1]
Pigeonpea sterility mosaic emaravirus 1	48.3 [CCP46989.1]	39.7 [YP_009237263.1]	38.0 [YP_009237281.1]	- ^a^	- ^a^
Blackberry leaf mottle-associated virus	48.2 [AQX45473.1]	40.1 [AQX45474.1]	38.3 [AQX45475.1]	37.4 [AQX45476.1]	- ^a^
Redbud yellow ringspot-associated emaravirus	48.0 [AEO95760.1]	39.2 [YP_009508087.1]	32.7 [YP_009508085.1]	23.4 [YP_009508084.1]	- ^a^
Fig mosaic emaravirus	48.0 [CAQ03479.7]	40.8 [YP_009237272.1]	40.5 [YP_009237270.1]	36.3 [YP_009237271.1]	14.3 [YP_009237275.1]
European mountain ash ringspot-associated emaravirus	47.9 [AAS73287.2]	36.4 [YP_003104765.1]	36.6 [YP_003104767.1]	7.7 [YP_003104766.1]	- ^a^
Rose rosette emaravirus	47.9 [ADZ54688.1]	41.2 [YP_004327590.1]	35.8 [YP_004327591.1]	36.6 [YP_004327592.1]	20.8 [YP_009380548.1]
Actinidia chlorotic ringspot-associated virus	47.8 [ALX00127.1]	37.8 [YP_009507926.1]	34.6 [YP_009507928.1]	25.1 [YP_009507927.1]	- ^a^
Raspberry leaf blotch emaravirus	32.4 [CBZ42024.1]	21.0 [YP_009237265.1]	18.7 [YP_009237266.1]	17.6 [YP_009237267.1]	11.6 [YP_009237278.1]
High Plains wheat mosaic emaravirus	30.2 [AIK23031.1]	21.0 [YP_009237256.1]	18.3 [YP_009237257.1]	18.6 [YP_009237258.1]	11.4 [YP_009237260.1]

^a^ Homologous protein sequences were not available in the NCBI database.

**Table 5:** Percent identity of protein sequence of Vitis cryptic virus with other *Partitiviridae*.

Genus	Virus	Vitis Cryptic Virus (Pool A;BCS51495.1/BCS51469.1)	
		RdRp [GenBank]	CP [GenBank]
Deltapartitivirus	Vitis cryptic virus (Pool B)	95.8 [BCS51497.1]	98.2 [BCS51498.1]
	Citrulluslanatus partitivirus	66.7 [APT68925.1]	47.5 [QLC27869.1]
	Pepper cryptic virus 1	65.7 [QEO60284.1]	39.7 [AVV48359.1]
	Pepper cryptic virus 2	59.9 [AVL84364.1]	37.9 [ALR34989.1]
	Beet cryptic virus 2	58.6 [QCF59322.1]	32.6 [QCF59321.1]
	Fig cryptic virus	37.7 [CBZ05548.1]	18.7 [YP_004429259.1]
Alphapartitivirus	White clover cryptic virus 1	19.6 [YP_086754.1]	4.5 [YP_086755.1]
Betapartitivirus	Atkinsonella hypoxylon virus	18.1 [NP_604475.1]	14.0 [NP_604476.1]
Crypsovirus	Cryptosporidium parvum virus 1	21.3 [YP_009508065.1]	16.8 [YP_009508066.1]
Gammapartitivirus	Penicillium stoloniferum virus S	19.7 [YP_052856.2]	1.8 [YP_052857.1]

## Data Availability

The sequence data have been submitted to the DDBJ/EMBL/GenBank databases under accession number LC602838-602841, LC604719-604732 and LC601811-601812. Addresses are as follows: GenBank http://www.ncbi.nlm.nih.gov.

## Supplementary Materials

The following are available online at https://www.mdpi.com/article/10.3390/v13050827/s1, Figure S1: Analysis of viral infections in *Vitis coignetiae*, Figure S2: Alignment of coat protein amino-acid sequence among Vitis emaravirus and other approved Emaravirus, Figure S3: Alignment of the coat protein amino-acid sequence among Vitis cryptic virus and other *Partitiviridae*, Figure S4: Some *Vitis coignetiae* samples used in this study showed viral-like mosaic symptoms, Table S1: Primer sequences used in this study.
